# The Development of Biocomposite Filaments for 3D Printing by Utilizing a Polylactic Acid (PLA) Polymer Matrix Reinforced with Cocoa Husk Cellulose Fibers

**DOI:** 10.3390/polym16131757

**Published:** 2024-06-21

**Authors:** Victor Hugo Martins de Almeida, Raildo Mota de Jesus, Gregório Mateus Santana, Sabir Khan, Erickson Fabiano Moura Sousa Silva, Iago Silva da Cruz, Ian de Souza Santos, Paulo Neilson Marques dos Anjos

**Affiliations:** 1 Department of Engineering and Computing, State University of Santa Cruz (UESC), Jorge Amado Highway, Km 16, Ilhéus 45662-900, Bahia, Brazil; efmssilva@uesc.br (E.F.M.S.S.); iagocruz.engmec@gmail.com (I.S.d.C.); iansouzasantos18@gmail.com (I.d.S.S.); 2 Department of Exact Sciences, State University of Santa Cruz (UESC), Jorge Amado Highway, Km 16, Ilhéus 45662-900, Bahia, Brazil; rmota@uesc.br (R.M.d.J.); gregorioengflorestal@gmail.com (G.M.S.); sabir_chemist@yahoo.com (S.K.); pauloneilson@uesc.br (P.N.M.d.A.)

**Keywords:** additive manufacturing, bioplastics, lignocellulosic materials, natural fiber, composites

## Abstract

Vegetable fibers are increasingly used in biocomposites, but there is a need for further development in utilizing by-products like cocoa husks. Three-dimensional printing, through Fused Filament Fabrication (FFF), is advancing rapidly and may be of great interest for applying biocomposite materials. This study focuses on developing innovative and fully biodegradable filaments for the FFF process. PLA filaments were prepared using cellulose fibers derived from cocoa husks (5% mass ratio). One set of filaments incorporated fibers from untreated husks (UCFFs), while another set utilized fibers from chemically treated husks (TCFFs). The fabricated materials were analyzed using scanning electron microscopy (SEM), thermogravimetric analysis (TGA), and Fourier transform infrared (FTIR) techniques, and they were also tested for tensile strength. ANOVA reveals that both UCFFs and TCFFs significantly predict tensile strength, with the UCFFs demonstrating an impressive R^2^ value of 0.9981. The optimal tensile strength for the filament test specimens was 16.05 MPa for TCFF8 and 13.58 MPa for UCFF8, utilizing the same printing parameters: 70% infill and a layer thickness of 0.10 mm. Additionally, there was an 18% improvement in the tensile strength of the printed specimens using the filaments filled with chemically treated cocoa husk fibers compared to the filaments with untreated fibers.

## 1. Introduction

Biocomposite materials of thermoplastic polymers reinforced with vegetable fibers have attracted the attention of researchers and companies seeking more sustainable alternatives with superior mechanical performance [1]. The mechanical strength properties of these biocomposites primarily depend on the type of incorporated vegetable fiber, the aspect ratio (length/diameter), the quality of adhesion at the interface between the embedded fiber and the polymer matrix, and the volume fraction of fiber added to the mixture [2]. While incorporating vegetable fibers into a polymer composite can improve its material properties, it can also worsen the properties of the material, depending on the interaction of the abovementioned factors. Beyond the mechanical properties, incorporating vegetable fibers can help reduce the consumption of pure polymer and make these materials more environmentally friendly. Research and development on and the application of these bio-based materials can benefit the ecosystem and contribute to local socio-economic development [3].

Several studies have shown the application of PLA together with a natural filler, such as bamboo, jute, flax, pineapple, sisal, or kenaf, to produce green composites [3,4,5]. In Kumar’s study [6], a tensile strength value of 61.07 MPa was reported for a PLA biocomposite with cellulose nanocrystals at proportions of 99/1%, respectively. Another notable study was presented by Liu [7] with a PLA biocomposite with sugarcane bagasse at proportions of 94/6%, respectively, which exhibited a tensile strength of 57.1 MPa. These studies have demonstrated that it is possible to produce biocomposites with optimized mechanical strength compared to pure PLA, serving as a reference to this study. 

Cocoa husks are highlighted in this research due to their local abundance and frequent disposal as waste from cocoa processing, making them a significant focus among lignocellulosic raw materials. Their utilization could offer sustainable solutions while addressing environmental concerns and contributing to local economic (*Theobroma cacao* L.) production; the husks represent approximately 80% of the total weight of the fruit. Cocoa fruit husks are produced in significant amounts within cocoa bean industries, yet they are often discarded due to their limited market value, despite being generated as waste in cocoa plantations [8]. Cocoa husks are a source of natural vegetable fiber, with lignocellulosic material comprising approximately 87% of the dry weight of the husks. Its chemical composition on a dry weight basis (%*w*/*w*) is approximately 35.4% cellulose, 37% hemicellulose, and 14.7% lignin, and the remainder consists of ash and other components, such as wax, pectin, moisture, impurities, etc. [9]. Therefore, pretreatments are essential in the cellulose extraction process and the removal of impurities.

This study outlines the development of biocomposite materials intended for use as raw materials (filament) in a 3D printer. Cocoa husk fibers were crushed, and cellulose microcrystals were chemically isolated. Two types of biocomposites (in the form of 3D filaments) were produced: one with untreated cocoa husk fibers and PLA and another with chemically isolated cellulose microcrystals and PLA. Alkaline and bleaching treatments were employed due to their recognized effectiveness in removing impurities from cellulose fibers, as well as them being more accessible and cost-effective techniques [10].

The amount of fiber added significantly impacts the mechanical properties of the composite. Studies indicate that the tensile strength tends to decrease, with a more pronounced reduction as the fiber content increases compared to the polymer. Overall, a lower fiber content tends to yield superior mechanical performance in biocomposites [8]. The fiber weight percentage added was 5% relative to PLA in both cases.

The untreated cocoa husk fibers and chemically treated microfibrils were analyzed using TGA and FTIR tests, while the mechanical properties of the printed parts with the filaments were tested using tensile tests. The mechanical tests followed international ASTM D638-14 [11]. 

## 2. Materials and Methods

### 2.1. Materials

PLA granules (Ingeo Biopolymer 3051D) suitable for 3D-printing the filaments, with a density of 1.24 g.cm ^3^, were used, sourced from NatureWorks^©^ (Minnetonka, MN, USA). Cocoa husks were purchased from a local market in Ilhéus, southern Bahia, Brazil. Sodium hydroxide (NaOH), sodium hypochlorite (NaClO), and glacial acetic acid from Sigma-Aldrich (Barueri, São Paulo, Brazil) were also used, available at the Laboratory of Materials and Environment (LAMMA) of the State University of Santa Cruz (UESC).

### 2.2. Processing of Cellulose Fibers

**Figure 1:**
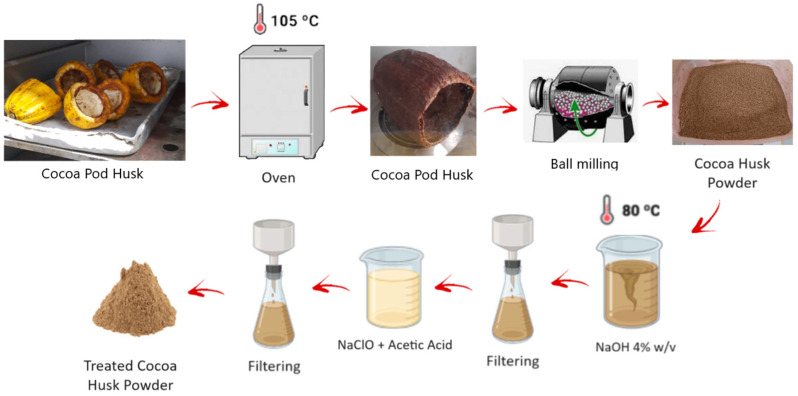
Processing steps for cocoa husk fibers.

The processing of the cocoa husks is summarized in Figure 1. The cocoa husks were washed to remove dirt and impurities and then dried at 105 °C in an oven until they reached a constant weight. Next, the pieces were ground using a knife mill. After grinding, the fiber was sieved through a mesh #325 (particles smaller than or equal to 44 µm). Part of the ground fibers, referred to as untreated cocoa husk fibers (UCF), was stored in a hermetic container for later use and analysis (chemical and morphological). The other part of the fibers was treated in a beaker with a 4% *w*/*v* sodium hydroxide (NaOH) solution at 80 °C for 4 h with mechanical stirring. After this treatment, the fibers underwent vacuum filtration, followed by washing with distilled water and then vacuum filtration again. Next, the fibers were treated in a 1:1 aqueous dilution of sodium hypochlorite (NaClO) for 30 min at 80 °C. Acetic acid was added until the solution reached a pH of 4. The fibers were then vacuum-filtered, washed with distilled water, dried, and finally ground in a ball mill for 60 min. The chemically treated fibers, referred to as treated cocoa husk fibers (TCFs), were stored in hermetic containers for later use and analysis.

### 2.3. Preparation of the Filaments and Printing of the Test Specimens

**Figure 2:**
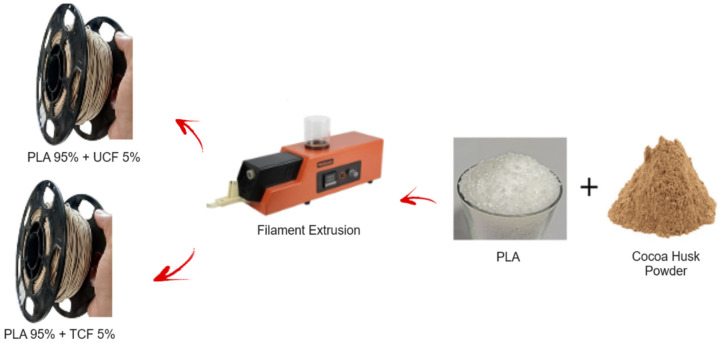
Extrusion steps for PLA with untreated cocoa husk fiber filament (UCFFs) and PLA with chemically treated cocoa husk fiber filaments (TCFFs).

Two types of filaments were created (Figure 2), one using untreated cocoa husk fiber, referred to as UCFF, and another with chemically treated cocoa husk fiber, called TCFF. In both cases, the cellulose microfibers and PLA pellets were weighed at a mass ratio of 5% fiber to 95% PLA and then dehumidified for 12 h at 60 °C. The filaments were manufactured using a single-screw extruder, where the fiber–PLA mixture was fed through a funnel to the extruder, and the heater was adjusted to temperatures between 185 °C and 205 °C. The filament diameter was 1.75 ± 0.05 mm.

**Figure 3:**
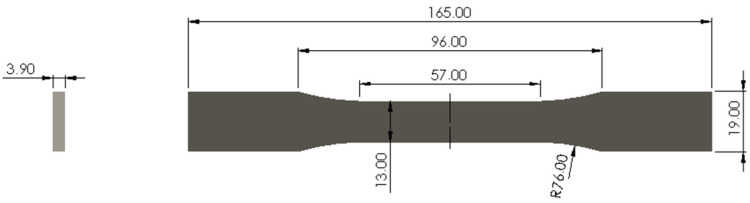
Type I test specimen for tensile strength testing according to ASTM D638-14.

The test specimens (TSs) printed for the tensile test were sized according to the guidelines of the international standard ASTM D638-14 [11]. The Type I TS used as a reference is illustrated in Figure 3.

The planning of the experiments, as well as the quantity of TSs manufactured by the 3D printing process, was defined using face-centered central composite design with two factors, two blocks, and ten experiments (Table 1).

The input variables chosen were infill percentage and layer height. For the infill percentage factor, a lower limit of 30%, a central point of 50%, and an upper limit of 70% were adopted. For the layer height factor, a lower limit of 0.1 mm, a central point of 0.15 mm, and an upper limit of 0.20 mm were adopted. These two variables of the 3D printing process were identified as critical for the analysis and will be the input parameters studied for comparison of the results in subsequent tests. The combinations of these parameters are detailed in Table 2.

The mechanical designs of the test specimens (TSs) were created using SolidWorks 2023© software, where they were also later exported into STL file format. These files were imported into the slicing software PrusaSlicer© 2.7.4, where the G-codes used in the 3D printer to manufacture each model were generated. The Fused Filament Fabrication (FFF) process parameters used in the Ender-3 Pro 3D printer (Creality, Shenzhen, China), at Laboratory of Mechanical Projects and Tribology (LAPMET) of UESC and configured in the PrusaSlicer© software for the manufacturing of the CPs are detailed in Table 3. These parameters were kept constant for all the test specimens to ensure standardization. The tensile tests were carried out in triplicate, totaling 30 printed test specimens for each of the two types of filaments.

### 2.4. Characterization Techniques for the Fibers and the Biocomposites 

A thermogravimetric (TGA) analyzer model DTG-60H (Shimadzu©, Barueri, São Paulo, Brazil) at the Laboratory of Research and Innovation in Advanced Materials (LAPIMA) of UESC was used to perform the thermogravimetric analysis on the untreated cocoa husk fibers (UCFs) and chemically treated cocoa husk fibers (TCFs). Approximately 6 mg of powder was placed in an alumina crucible and heated to 1000 °C at an incremental rate of 10 °C·min^−1^ under a nitrogen atmosphere with a flow rate of 100 mL·min^−1^. 

The Fourier transform infrared (FT-IR) spectra of the untreated cocoa husk fibers (UCFs) and chemically treated cocoa husk fibers (TCFs) were recorded using an Attenuated Total Reflectance (ATR) spectrometer, model IRPrestige-21 (Shimadzu©), at the Laboratory of Research and Innovation in Advanced Materials (LAPIMA) of UESC. The spectra were obtained in the wavenumber range between 4000 and 450 cm^−1^.

The morphology of the untreated cocoa husk fibers (UCFs) and chemically treated cocoa husk fibers (TCFs) was visualized using a Quanta 250 Scanning Electron Microscope (SEM), model (FEI Company©, Hillsboro, OR, USA), at the Center for Electron Microscopy (CME) of UESC. The powdered materials were placed on double-sided carbon adhesive tape in the sample holder. The sample was gently pressed with paper, and the excess material was removed. Subsequently, the morphological characteristics were visualized.

The tensile strength mechanical tests were conducted according to the guidelines of the international ASTM standards. For the tensile test, the reference was ASTM standard D638-14 [11]. The Instron 5982 universal testing machine (Instron©, Norwood, MA, USA) was used to perform the tensile test, with a crosshead speed of 0.0000254 m.s^−1^ (1.524 mm.min^−1^) at the Laboratory of Experimental Measurements and Uncertainty Assessment (LAMEAI) of UESC.

### 2.5. Statistical Analyses

Response Surface Methodology (RSM) was employed to investigate the relationship between tensile strength (σt) and the parameters of internal infill and layer height. The experimental data were analyzed using Excel 365© and Minitab 21.3.0 © software. Analysis of variance (ANOVA) was conducted to determine the significance level of each factor (linear, quadratic, and a linear combination of these factors).

A significance level of 95% was adopted, corresponding to a *p*-value of ≤0.05. According to [12], there are five indices for validating a model: the coefficient of determination (R^2^), mean absolute percentage error (MAPE), root mean square error (RMSE), relative error (%), and multi-objective error function (MO).

The R^2^ and RMSE indices were used. There are no specific values for each of these indices; however, for R^2^, the higher the percentage value, the greater the influence of the analyzed variables on the response. RMSE evaluates the performance of prediction models by providing a measure of how well the predictions match the observed data. A lower RMSE value indicates a better fit of the model to the observed data, with a value near zero indicating a closer match between the observed and predicted values [12].

## 3. Results

### 3.1. Characterizations Analysis

#### 3.1.1. Morphological Characterization

**Figure 4:**
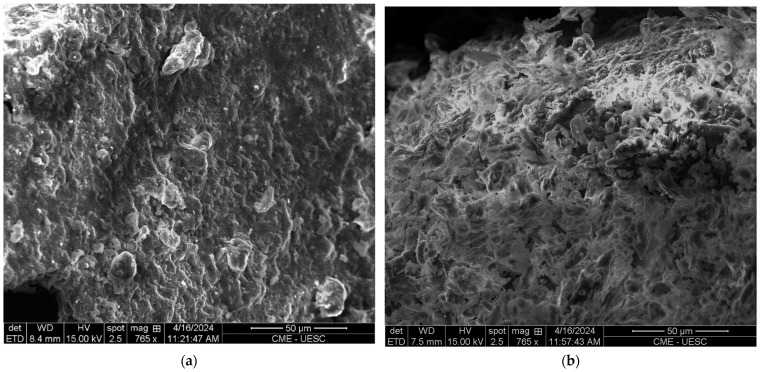
SEM images of (**a**) the cellulose fibers from untreated cocoa husk (UCFs); and (**b**) of the cellulose fibers from the chemically treated cocoa husk (TCFs).

A detailed comparative analysis of the surface morphologies of both the untreated cocoa husk fiber samples and those chemically treated is depicted in detail in Figure 4 using scanning electron microscopy (SEM). Figure 4a shows a SEM micrograph of the untreated cocoa husk fibers (UCFs), while Figure 4b displays a SEM micrograph of the cocoa husk fiber samples subjected to chemical treatment (TCFs). Analyzing the surface morphology of the fibers was pivotal to discern the surface charge of these samples, especially in the case of the untreated fibers. In Figure 4a, it is evident that the surface of the untreated cocoa husk fiber (UCF) exhibits greater uniformity, forming a denser and more filled material due to the presence of waxes, hemicellulose, pectin, lignin, and other impurities [13]. Conversely, in Figure 4b, the surface of the chemically treated cocoa husk fiber (TCF) displays increased porosity, with more empty spaces between the material, attributed to the partial or complete removal of waxes, hemicelluloses, pectin, and lignin.

**Figure 5:**
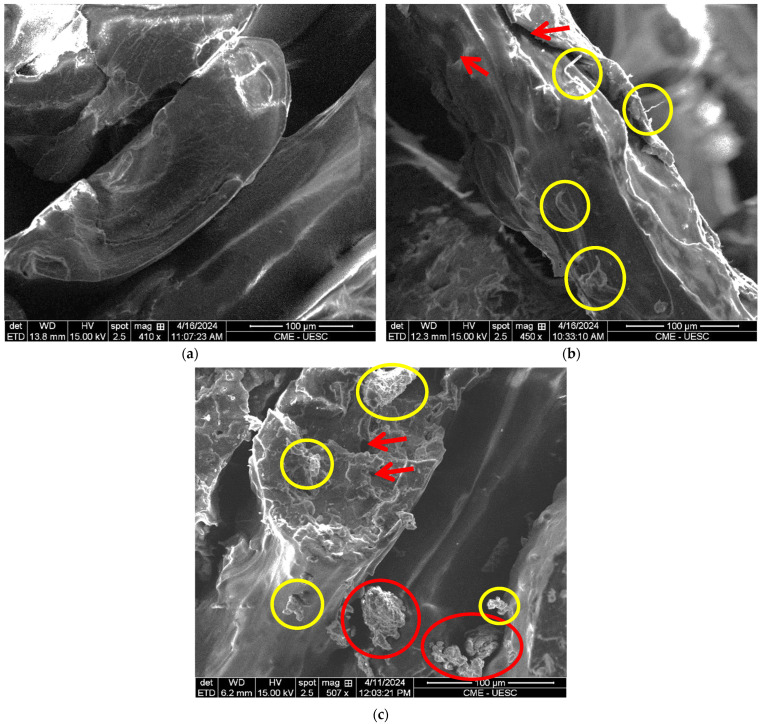
SEM images of fractured specimens after tensile testing: (**a**) pure PLA specimen; (**b**) PLA specimen with untreated cocoa husk cellulose fibers (UCFFs); (**c**) PLA specimen with chemically treated cocoa husk cellulose fibers (TCFFs).

The SEM micrographs of the fractured test specimens of pure PLA, the test specimens created with filament using untreated cocoa husk fiber (UCFF), and test specimens created with filament using cocoa husk fiber subjected to chemical treatment (TCFF) are presented in Figure 5. The basic goal was to determine whether there were any observable fiber aggregates on the fractured surfaces. In comparison to pure PLA (Figure 5a), the surfaces of the UCFF (Figure 5b) and TCFF (Figure 5c) biocomposites exhibited rougher textures, with protrusions, small voids (red arrows), and well-dispersed fibers (yellow circles). In the TCFF specimen (Figure 5c), areas attributed to small fiber aggregates (red circles) can be observed, which are only visible in the TCFF sample. However, overall, a good dispersion of fibers was achieved in the UCFF and TCFF biocomposites.

#### 3.1.2. Thermogravimetric Analysis

**Figure 6:**
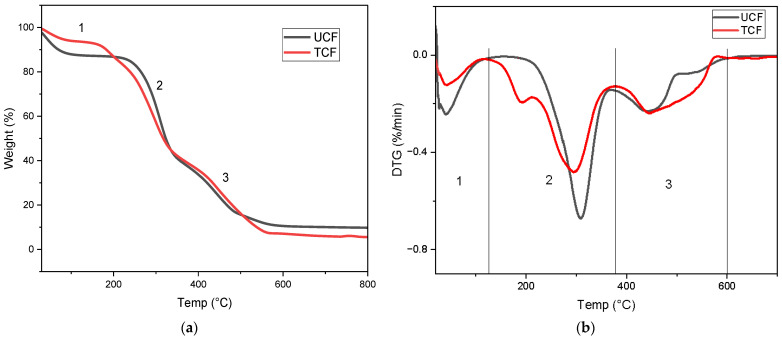
TGA (**a**) and DTG (**b**) graphs of cellulose fibers from untreated cocoa husks (UCF) and cellulose fibers from chemically treated cocoa husks (TCF).

The thermogravimetric analysis of untreated cocoa husk fiber (UCF) and chemically treated cocoa husk fiber (TCF) is presented in Figure 6a. The TGA plot illustrates the percentage of mass loss concerning the temperature increase. Both samples exhibit distinct stages of mass loss in the thermogravimetric analysis, indicating the thermal degradation behavior of untreated and chemically treated cocoa husk fibers. A marginal decrease in mass percentage is observed in the samples from 20 °C to 110 °C, with the UCF displaying the highest mass loss in this range, around 14%, and the TCF showing the lowest loss, approximately 6%. The decline in mass percentage observed in this range is attributed to the evaporation of moisture adsorbed on the sample surfaces, along with chemisorbed water molecules bound to the hydrogen within the samples [13,14]. The second region was identified between 130 and 380 °C in the TCF sample and between 210 and 380 °C in the UCF sample. This range corresponds to the degradation primarily of hemicellulose and cellulose, along with a fraction of the lignin. The third region of thermal degradation occurs at temperatures between 380 and 600 °C. Temperatures above 380 °C are associated with the pyrolysis of lignin [15] and the oxidation of the fixed carbon present in cocoa husks [13].

Beyond 600 °C, ash formation constitutes the final residue, comprising inorganic elements. The untreated cocoa husk fiber (UCF) and chemically treated fiber (TCF) left residues of 9.8% and 5.5% of the initial mass, respectively.

The DTG curves (Figure 6b) of TCF and UCF revealed cellulose degradation peaks at 295 and 309 °C, respectively. TCF exhibited a maximum thermal stability temperature of 295 °C, lower than UCF due to the reduced cellulose crystallinity [9], as confirmed by the FTIR spectral analyses. TCF exhibited a peak at 190 °C, attributed to the residual hemicellulose [12]. Above 380 °C, a continuous and slow weight loss is attributed to lignin degradation [13], with a degradation peak at 440 °C for both UCF and TCF.

#### 3.1.3. Fourier Transform Infrared Spectroscopy (FTIR)

**Figure 7:**
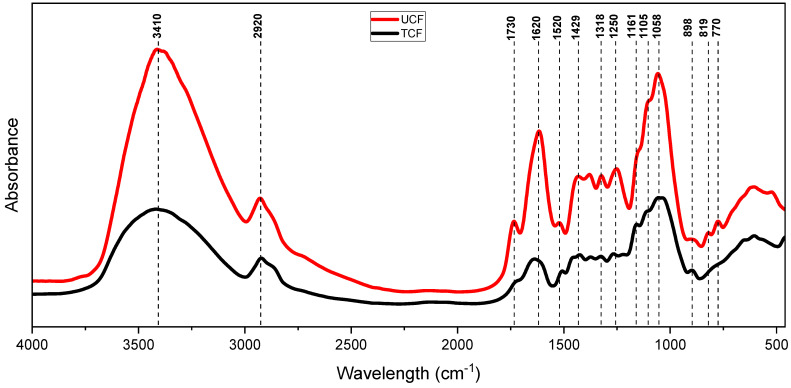
FTIR spectra of untreated cocoa husk fiber (UCF) and chemically treated cocoa fiber (TCF).

The change in the chemical composition of chemically treated cocoa husk fiber (TCF) relative to untreated cocoa husk fiber (UCF) is illustrated in the FTIR spectrum according to the absorbance in Figure 7. Through this analysis, the components of the raw material were verified due to the existence of the principal bands related to cellulose, hemicellulose, and lignin. While it is a qualitative analysis, the peak amplitudes can indicate the substance concentration or the number of molecules with specific vibrations. Thus, the differences in the amplitudes of the spectra between UCF and TCF are indicative of the removal of lignin, hemicellulose, and extractives.

The spectral region between 3200 and 3600 cm^−1^ indeed corresponds to the stretching vibration of the hydroxyl (OH) group band, which is commonly found in biopolymers such as cellulose, lignin, and hemicellulose. Additionally, this peak includes inter- and intramolecular hydrogen bonding vibrations in cellulose [13].

With the removal of part of the lignin and hemicellulose, this peak tended to become narrower and sharper [16] since the TCF was also milled in the ball mill, which caused some crystalline cellulose structures to break, resulting in a more amorphous structure; a reduction in signal intensity at 3410 cm^−1^ can be observed. These results are consistent with literature reports on the effects of mechanical milling on the structure of cellulose [17]. 

The peak at 2920 cm^−1^, more prominent in the UCF spectrum, corresponds to the stretching vibrations of C-H from the methyl and methylene groups of lignin and hemicellulose [16,18]. The reduction observed in the TCF indicates the partial removal of these components. The peak at 1730 cm^−1^ corresponds to the C=O stretching vibrations of the acetyl and ester groups found in hemicellulose and fiber extractives [19]. It is solely present in UCF, suggesting the removal of these components in TCF. The peak at 1620 cm^−1^, attributed to the aromatic ring of lignin [20], can be observed in both spectra, with a lower intensity in TCF, indicating once again the partial removal of lignin. Similarly, the reduction in the bands at 1520 cm^−1^ and 1250 cm^−1^, related to C=C group and C-H bond vibrations in lignin’s methylene and methyl groups [21,22], also corroborates these results.

The absorption band at 1429 cm^−1^ corresponds to the asymmetric stretching of CH_2_, indicating the crystallinity of the cellulose material. This peak becomes broader if the cellulose material has lower crystallinity [23]. In the case of TCF, this signal is broader, which corroborates the decrease in the crystallinity of the cellulose present, as the TCF samples were milled in a ball mill.

A band at 1318 cm^−1^ corresponds to the deformation of O-H and the vibration of CH2 in cellulose [24]. The vibration at 1161 cm^−1^ is attributed to the anti-symmetric stretching vibration of the C-O-C bridge, while the vibration at 1105 cm^−1^ is designated for the anti-symmetric stretching band of the in-plane ring. The intensity of these two peaks also decreases as the crystallinity of cellulose decreases [23]. The band at 1058 cm^−1^ has been associated with the stretching of C-C and C-O in polysaccharides and lignin [16,17,25]; there was a reduction in signal intensity in TCF, corroborating the removal of hemicellulose and lignin during the chemical treatment.

The vibration at 898 cm^−1^ results from the stretching of C-O-C in the β-(1-4) glycosidic linkage. This band is identified as the absorption band of amorphous cellulose, whose relative intensity increases with a reduction in crystallinity [17,23,26]. There was an increase in this peak in TCF due to the increase in the amorphous phase of cellulose after milling in the ball mill.

In UCF, there are peaks in the region near 819 cm^−1^ and 770 cm^−1^, which have been associated with the functional groups of the deformed C-H bonds present in lignin [27,28]; there was a significant reduction in these bands in TCF, confirming the removal of part of the lignin after the chemical treatments applied to the raw fibers.

### 3.2. Results of Mechanical Tensile Tests

The results from the mechanical tensile test for the PLA/cocoa husk fiber biocomposite filaments are presented in Table 4. The plot shows the average values of tensile strength (σt), modulus of elasticity (Et), and strain (ε%) for the PLA filament with untreated cocoa husk fibers (UCFF) and the PLA filament with chemically treated cocoa husk fiber (TCFF). The best tensile strength (σt) result for the printed specimen using TCFF filaments was 16.05 MPa (TCFF8), while for the UCFF filament specimen, it was 13.58 MPa (UCFF8), both printed with the same settings: 70% infill and a 0.10 mm layer thickness.

This comparison highlights a remarkable 18% enhancement in tensile strength for the TCFF filaments, suggesting that the chemical treatment applied to the fibers had a significant impact on the mechanical property of tensile strength. However, when comparing the tensile strength of the TCFF8 filaments at 16.05 MPa with the pure PLA filaments reported by [29] with σt = 29.5 MPa, we observe a decrease of approximately 45% in tensile strength for the TCFFs.

The highest elastic modulus (Et) values recorded for the test specimens printed with the filaments were TCFF8 = 542.4 MPa and FFCN8 = 474.8 MPa, respectively. These figures indicate that the FFCQ filaments demonstrated greater rigidity compared to FFCN under the specific conditions of the tensile test, emphasizing the positive effect of chemical treatment on the material’s elastic properties.

The analysis of variance (ANOVA) for tensile strength yielded an R^2^ = 0.9981 and an RMSE = 0.07079 for UCFF and an R^2^ = 0.9979 and an RMSE = 0.08482 for TCFF. With an R^2^ = 0.9981 for the variable (UCFF), this suggests that roughly 99.81% of the variation in tensile strength can be explained by the statistical model used, indicating a strong and significant relationship between the independent and dependent variables in this context. The R^2^ = 0.9979 for (TCFF) suggests that approximately 99.79% of the variability in tensile strength is explained by the corresponding model for this variable. Both results indicate a strong relationship between the independent variables (infill and layer height) and tensile strength. Considering the stress data scale in MPa, the RMSE is relatively small in both cases, suggesting that the prediction errors are proportionate to the inherent variability of the data. These findings imply that the model holds significance and statistical significance.

**Figure 8:**
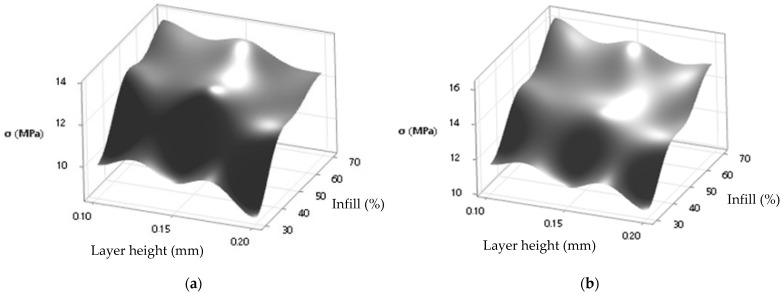
Surface response plot for tensile strength: σt (MPa) × layer height (mm)/infill (%) for (**a**) UCFF filament specimens and (**b**) TCFF filament specimens.

Based on the obtained data and the results of the ANOVA for tensile strength, it was possible to establish a model of tensile strength (σt) as a function of the parameters percentage of infill (%) and layer height (mm). Figure 8a illustrates the response surface plot for the UCFF material, whereas Figure 8b shows the response surface plot for the TCFF material.

## 4. Discussion

Biocomposites with PLA granules and cellulose fibers from cocoa husks were premixed and compounded by extrusion in an attempt to create filaments for 3D printing. The results obtained in this study demonstrated the feasibility of using this agricultural waste as reinforcement in PLA biocomposites, applied as raw material in the filament production industry for 3D printers.

FTIR and TGA analyses revealed significant differences in the structural and thermal characteristics of the cocoa husk fiber samples. The presence of bands related to cellulose was confirmed in both samples. Additionally, reductions were observed in bands related to water molecules, low-molecular-weight organic compounds, metallic oxides, lignin, and hemicellulose in the chemically treated cocoa husk fibers (TCF). Thermal degradation changes between UCF and TCF were evident, corroborating the structural changes indicated in the FTIR analyses. The analysis highlights the effectiveness of chemical treatment in modifying the thermal and structural properties of cocoa husk fibers.

Morphological studies of cocoa husk fiber and the generated biocomposites showed good fiber dispersion in PLA; however, small aggregates occurred in TCFF, indicating that the fibers were not as well dispersed in this composite.

The mechanical tests demonstrated a superior performance by 18% in terms of the tensile strength of the PLA filament specimens with cellulose fibers from cocoa husks subjected to chemical treatment (TCFFs) compared to filaments containing untreated fibers (UCFFs). This analysis highlights the significant influence of chemical treatment on fibers for enhancing biocomposite performance. However, when comparing the tensile strength result for the pure PLA filament specimens with that of the TCFF filament specimens, it was found that the incorporation of fibers resulted in a reduction of approximately 45% in tensile strength properties. Although there was a decrease in tensile strength, the study highlights the importance of investigating variations in the manufacturing process to understand their impact on composite performance better. Additionally, a significant improvement in quality was observed when the fibers were treated. However, other aspects of the manufacturing process, such as the bonding between the fibers and PLA, the optimal fiber quantity, and the possibility of adding additives to enhance the composite properties, also require investigation to optimize performance and applicability.

In all cases, printing settings with a 70% infill density and a layer height of 0.10 mm yielded the best results. This configuration demonstrated an effective combination of high strength, material efficiency, and printing speed, making it a favorable choice. It is worth noting that balancing the desired strength with practical considerations such as print time and overall process efficiency is important for optimizing part performance in real-world contexts. These results provide valuable insights for the optimization of 3D printing processes, as they indicate which specific settings can result in the desired mechanical properties.

This study demonstrates that the process of creating biocomposites with cocoa husk fibers is a promising method; however, further research is needed to improve the dispersion and adhesion of fibers in PLA.

## Figures and Tables

**Table 1:** Levels of adjustment for input variables: infill (%) and layer height (mm) for central composite design, face-centered.

Factor	Lower Limit (−1)	Central Point (0)	Upper Limit (+1)
Infill (%)	30	50	70
Layer height (mm)	0.10	0.15	0.20

**Table 2:** Central composite design—central face, experiments for the input variables infill and layer height.

Test Specimen (TS)	Infill (%)	Layer Height (mm)
01	30	0.10
02	30	0.15
03	30	0.20
04	50	0.10
05	50	0.15
06	50	0.15
07	50	0.20
08	70	0.10
09	70	0.15
10	70	0.20

**Table 3:** FFF process parameters used in the printing of the TSs.

Parameter	Infill (%)
Extruder Diameter (mm)	0.40
Extrusion Multiplier	1.10
Layer Height	As per Table 2
Number of Top Layers	4
Number of Bottom Layers	4
Number of Perimeter Lines	3
Internal Infill Pattern	Hexagonal
External Infill Pattern	Rectilinear
Infill (%)	As per Table 2
Internal Infill Angle (°)	45
Extrusion Temperature (°C)	180
Bed Temperature (°C)	60
Extruder Travel Speed (mm/s)	40
Filament Diameter (mm)	1.75

**Table 4:** Tensile strength values (σt), elastic modulus (Et), and deformation (ε%) for test specimens printed with PLA filaments containing untreated cocoa husk fibers (UCFFs) and PLA filaments containing chemically treated cocoa husk fibers (TCFFs).

Test Specimen (TS)	Infill (%)	Layer Height (mm)	Tensile Strength (MPa)	Elastic Modulus (MPa)	Strain (ε%)
UCFF1	30	0.10	10.08	358.6	2.82
UCFF2	30	0.15	9.62	322.9	2.99
UCFF3	30	0.20	8.67	279.8	3.11
UCFF4	50	0.10	12.95	466.5	2.81
UCFF5	50	0.15	12.77	442.7	2.89
UCFF6	50	0.15	12.69	436.3	2.92
UCFF7	50	0.20	11.54	379.9	3.05
UCFF8	70	0.10	13.58	474.8	2.88
UCFF9	70	0.15	13.33	449.5	2.99
UCFF10	70	0.20	12.43	389.8	3.19
TCFF1	30	0.10	11.56	382.5	3.02
TCFF2	30	0.15	10.98	336.7	3.27
TCFF3	30	0.20	10.36	306.5	3.39
TCFF4	50	0.10	14.34	485.3	2.96
TCFF5	50	0.15	13.56	446.4	3.04
TCFF6	50	0.15	13.70	446.1	3.08
TCFF7	50	0.20	12.89	408.0	3.16
TCFF8	70	0.10	16.05	542.4	2,96
TCFF9	70	0.15	15.70	511.8	3.07
TCFF10	70	0.20	14.96	474.1	3.16

## Data Availability

The original contributions presented in the study are included in the article, further inquiries can be directed to the corresponding author.

## References

[1] Inseemeesak B., Siripaiboon C., Somkeattikul K., Attasophonwattana P., Kiatiwat T., Punsuvon V., Areeprasert C. (2022). Biocomposite Fabrication from Pilot-Scale Steam-Exploded Coconut Fiber and PLA/PBS with Mechanical and Thermal Characterizations. J. Clean. Prod..

[2] Das A.K., Agar D.A., Rudolfsson M., Larsson S.H. (2021). A Review on Wood Powders in 3D Printing: Processes, Properties and Potential Applications. J. Mater. Res. Technol..

[3] Dai L., Cheng T., Duan C., Zhao W., Zhang W., Zou X., Aspler J., Ni Y. (2019). 3D Printing Using Plant-Derived Cellulose and Its Derivatives: A Review. Carbohydr. Polym..

[4] Kopparthy S.D.S., Netravali A.N. (2021). Review: Green Composites for Structural Applications. Compos. Part C Open Access.

[5] Shekar H.S.S., Ramachandra M. (2018). Green Composites: A Review. Mater. Today Proc..

[6] Kumar S.D., Venkadeshwaran K., Aravindan M.K. (2020). Fused Deposition Modelling of PLA Reinforced with Cellulose Nano-Crystals. Mater. Today Proc..

[7] Liu H., He H., Peng X., Huang B., Li J. (2019). Three-dimensional Printing of Poly(Lactic Acid) Bio-based Composites with Sugarcane Bagasse Fiber: Effect of Printing Orientation on Tensile Performance. Polym. Adv. Technol..

[8] Vásquez Z.S., De Carvalho Neto D.P., Pereira G.V.M., Vandenberghe L.P.S., De Oliveira P.Z., Tiburcio P.B., Rogez H.L.G., Neto A.G., Soccol C.R. (2019). Biotechnological Approaches for Cocoa Waste Management: A Review. Waste Manag..

[9] Daud Z., Awang H., Mohd Kassim A.S., Mohd Hatta M.Z., Mohd Aripin A. (2014). Cocoa Pod Husk and Corn Stalk: Alternative Paper Fibres Study on Chemical Characterization and Morphological Structures. Adv. Mater. Res..

[10] Almeida V.H.M., Jesus R.M., Santana G.M., Pereira T.B. (2024). Polylactic Acid Polymer Matrix (Pla) Biocomposites with Plant Fibers for Manufacturing 3D Printing Filaments: A Review. J. Compos. Sci..

[11] (2022). Standard Test Method for Tensile Properties of Plastics.

[12] Myers R.H., Montgomery D.C., Anderson-Cook C.M. (2016). Response Surface Methodology: Process and Product Optimization Using Designed Experiments.

[13] Akinjokun A.I., Petrik L.F., Ogunfowokan A.O., Ajao J., Ojumu T.V. (2021). Isolation and Characterization of Nanocrystalline Cellulose from Cocoa Pod Husk (CPH) Biomass Wastes. Heliyon.

[14] Yu W., Dong L., Lei W., Zhou Y., Pu Y., Zhang X. (2021). Effects of Rice Straw Powder (RSP) Size and Pretreatment on Properties of FDM 3D-Printed RSP/Poly(Lactic Acid) Biocomposites. Molecules.

[15] Díaz-Oviedo A.F., Ramón-Valencia B.A., Moreno-Contreras G.G. (2022). Caracterización Físico-Química de La Cáscara de Mazorca de Cacao Como Posible Uso En La Elaboración de Tableros Aglomerados. Rev. Investig. Desarro. E Innov..

[16] Hozman-Manrique A.S., Garcia-Brand A.J., Hernández-Carrión M., Porras A. (2023). Isolation and Characterization of Cellulose Microfibers from Colombian Cocoa Pod Husk via Chemical Treatment with Pressure Effects. Polymers.

[17] Agarwal U.P., Ralph S.A., Baez C., Reiner R.S. (2021). Contributions of Crystalline and Noncrystalline Cellulose Can Occur in the Same Spectral Regions: Evidence Based on Raman and IR and Its Implication for Crystallinity Measurements. Biomacromolecules.

[18] Gao X., Jia Y., Chen Z., Santhanam R.K., Zhang M., He C., Chen H. (2022). Synthesis of Hydrogels Based on Nanocellulose from Garlic Straw and Regulating the Release of Allicin and Its Cytotoxicity. Food Sci. Technol..

[19] Garcia-Brand A.J., Morales M.A., Hozman A.S., Ramirez A.C., Cruz L.J., Maranon A., Muñoz-Camargo C., Cruz J.C., Porras A. (2021). Bioactive Poly(Lactic Acid)–Cocoa Bean Shell Composites for Biomaterial Formulation: Preparation and Preliminary In Vitro Characterization. Polymers.

[20] Kaur V., Dash B.P., Vermani S., Devi A. (2022). Extraction, Quantification and Characterization of Lignin Extracted from Bamboo Biomass. Mater. Today Proc..

[21] Kłosowski G., Mikulski D. (2023). Changes in Various Lignocellulose Biomasses Structure after Microwave-Assisted Hydrotropic Pretreatment. Renew. Energy.

[22] Veber A., Zancajo V.M.R., Puskar L., Schade U., Kneipp J. (2023). In Situ Infrared Imaging of the Local Orientation of Cellulose Fibrils in Plant Secondary Cell Walls. Analyst.

[23] Dassanayake R.S., Fierro J.S., Abidi N., Quitevis E.L., Boggavarappu K., Thalangamaarachchige V.D. (2023). Characterization of Cellulose Nanocrystals by Current Spectroscopic Techniques. Appl. Spectrosc. Rev..

[24] Popescu C.-M., Larsson P.T., Olaru N., Vasile C. (2012). Spectroscopic Study of Acetylated Kraft Pulp Fibers. Carbohydr. Polym..

[25] Shi J., Xing D., Lia J. (2012). FTIR Studies of the Changes in Wood Chemistry from Wood Forming Tissue under Inclined Treatment. Energy Procedia.

[26] Oh S.Y., Yoo D.I., Shin Y., Seo G. (2005). FTIR Analysis of Cellulose Treated with Sodium Hydroxide and Carbon Dioxide. Carbohydr. Res..

[27] Eugenio M.E., Martín-Sampedro R., Santos J.I., Wicklein B., Ibarra D. (2021). Chemical, Thermal and Antioxidant Properties of Lignins Solubilized during Soda/AQ Pulping of Orange and Olive Tree Pruning Residues. Molecules.

[28] Bhagia S., Ďurkovič J., Lagaňa R., Kardošová M., Kačík F., Cernescu A., Schäfer P., Yoo C.G., Ragauskas A.J. (2022). Nanoscale FTIR and Mechanical Mapping of Plant Cell Walls for Understanding Biomass Deconstruction. ACS Sustain. Chem. Eng..

[29] Mansingh B.B., Binoj J.S., Tan Z.Q., Eugene W.W.L., Amornsakchai T., Hassan S.A., Goh K.L. (2022). Comprehensive Characterization of Raw and Treated Pineapple Leaf Fiber/Polylactic Acid Green Composites Manufactured by 3D Printing Technique. Polym. Compos..

